# Cell differentiation in the vascular cambium: new tool, 120-year debate

**DOI:** 10.1093/jxb/ery285

**Published:** 2018-08-13

**Authors:** Ana C Ramos, Sharon Regan

**Affiliations:** Department of Biology, Queen’s University, Kingston, Ontario, Canada

**Keywords:** Cambium initials, cell fate, poplar, stem cells, wood formation, xylogenesis

## Abstract

This article comments on:

Bossinger G, Spokevicius AV. 2018. Sector analysis reveals patterns of cambium differentiation in poplar stems. Journal of Experimental Botany **69,** 4339–4348.

**Poplar is an important model for gene discovery and characterization. These trees undergo extensive secondary growth to produce wood from the vascular cambium, but it has been challenging to visualize the earliest stages of development. Bossinger and Spokevicius (2018) have used a technique known as sector analysis to visualize cell fate, addressing a long-standing debate on the origins of xylem and phloem in tree cells and providing an unprecedented look at initial wood formation.**

*Populus* spp., commonly called cottonwoods, aspens or poplars, inhabit much of the northern hemisphere yielding up to 19 Mg ha^–1^ year^–1^ of biomass in Canada and the United States alone (Sannigrahi *et al.*, 2010). Having a sequenced genome (Tuskan *et al.*, 2006), numerous studies of global gene expression (e.g. Schrader *et al.*, 2004) and collections of activation- (reviewed by Busov *et al.*, 2010) and transposon-tagged (Fladung, 2011) mutants, they are an important model for gene discovery and characterization in angiosperm trees. Unlike other model plants such as Arabidopsis, *Populus* spp. are perennial trees that undergo extensive secondary growth to produce wood involving the vascular cambium (a lateral meristem). Bossinger and Spokevicius (2018) have used a technique known as sector analysis to visualize cell fate in the vascular cambium and provide an unprecedented look at the early stages of wood development.

## *Populus* wood and the importance of secondary growth

*Populus* wood is an important feedstock for the pulp and paper industry (Christersson, 2008), and having a heating value of 19 MJ kg^–1^ for hybrid species, it plays an important role in bioenergy production through thermochemical conversion (e.g. gasification, pyrolysis, hydrothermal liquefaction) (reviewed by Sannigrahi *et al.*, 2010; Slopiecka *et al.*, 2012; Chen *et al.*, 2016; Tekin *et al.*, 2016). Additionally, *Populus* wood offers an excellent platform for biorefinery development, since the biomass is rich in sugars that can be extracted and used to produce fermentation products such as bioethanol and polyhydroxybutyrates (Dai and McDonald, 2014; Demartini *et al.*, 2015). Thus, key components of secondary xylem, mainly cellulose, hemicellulose and lignin, have become the focus of many studies, but our knowledge of the early stages of wood development remains limited.

The vascular cambium is a cylindrical meristem that gives rise to xylem (wood) and phloem during secondary growth. While there is plenty of knowledge on the function and differentiation of primary growth meristems (apical and root meristems) based on studies in annual plants such as Arabidopsis, differentiation of the vascular cambium during secondary growth is still poorly understood despite the important role of this meristem in producing secondary xylem and phloem. One of the challenges of studying cells in the cambial zone is the difficulty in morphologically distinguishing cambial initials from mother cells and young xylem and phloem, making it hard to develop a model to describe division and differentiation patterns. What we know of vascular cambium differentiation is that cambial initials have the capacity to give rise to both xylem and phloem mother cells, which in turn give rise to xylem and phloem elements (Box 1). However, there has been a long-standing debate as to whether mother cells are derived from a single layer of cambial initials, or whether multiple layers of cambial initials exist across several radial files (starting with Sanio, 1873, and Raatz, 1892).

**Box 1. Structure of the cambial zone**

There are two zones that make up the vascular cambium: the division zone and the differentiation zone. The division zone is where the cambial initials undergo cell division, which can occur parallel to, or perpendicular to, the surface of the closest organ (periclinal or anticlinal cell division, respectively). After cambial initials divide, they enter the differentiation zone, forming either xylem or phloem mother cells and, in turn, xylem or phloem. Bossinger and Spokevicius (2018) used sector analysis to determine that cambial initials originate from a single layer of cells, adding compelling evidence to a 120-year debate.

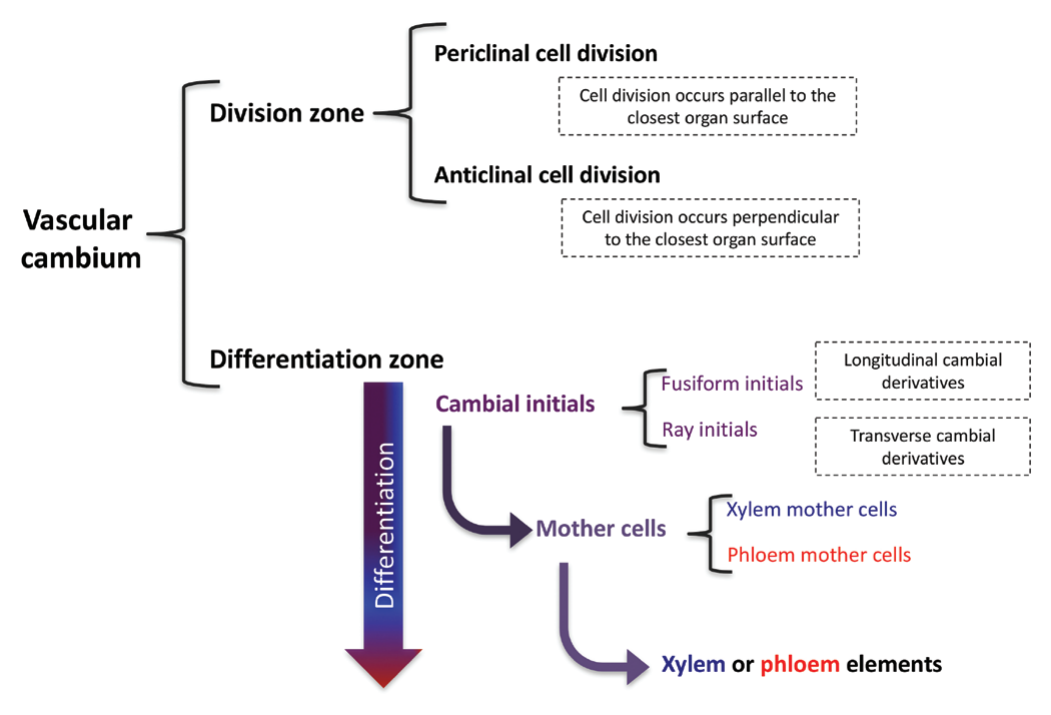

Along with the growing number of genomic and proteomic resources available for *Populus* has come a growing list of genes responsible for wood development and secondary growth with a major focus on cambial meristem activity, and wood quality based on cell wall properties (e.g. Obudulu *et al.*, 2016; Kucukoglu *et al.*, 2017; Li *et al.*, 2017). Despite this, there is a need for a robust way to visualize cell differentiation in a mature stem to follow xylem and phloem development from the cambial initials and mother cells on to mature tissue.

## Sector analysis reveals cell fate in the cambial zone

Bossinger and Spokevicius have developed a system called sector analysis which uses the β-glucuronidase (GUS) reporter gene to follow cell fate in the tree stem. Cells are transformed in the cambial zone of a living tree and, after several months of subsequent growth, the cell and any of its derivatives are revealed by histochemical GUS staining. After creating hundreds of independent transformation events of cells in the cambial zone, this study provides a high-throughput and unprecedented look at the differentiation of cambial initials and mother cells into xylem and phloem. Their evidence provides strong support for three major findings: (i) the vascular cambium is made up of a single layer of cambial initials, (ii) the differentiation of both phloem and xylem mother cells is controlled independently, and (iii) that on average four xylem cells are produced for every one phloem cell (ranging from 2:1 to 6:1). Unlike previous studies that proposed models for the differentiation of the vascular cambium based on anatomical analysis of past cell divisions, sector analysis labels a single cell and follows the fate of its derivatives throughout the stages of xylem and phloem development. This opens up the opportunity to analyse gene function in the cambial zone at a much higher resolution than previously possible.

## Future directions

The research by Bossinger and Spokevicius (2018) not only answers basic questions about cambial meristem cell fate and differentiation in *Populus*, but also provides a standard framework of wild-type cell differentiation in the tree stem. In our own research using activation tagged *Populus* (Harrison *et al.*, 2007), we have identified several mutants with alterations in wood development including the ratio of xylem to phloem, wood biomass (which is increased) and altered wood properties such as lignin content. Testing these mutants with sector analysis would enable a closer look at the impact of these mutations on the vascular cambium and cell differentiation in the stem, and bring us closer to understanding the intricacies of gene function. Similarly, other studies of candidate genes involved in cambium development or wood formation that use either natural variants or transgenic lines for functional analysis could benefit from incorporating sector analysis to resolve the impact of genes on cell differentiation at the level of individual cell lineages as previously shown by Spokevicius *et al.* (2016). This is certainly a valuable addition to the *Populus* tool box for functional characterization of wood formation.
